# Rapid transcriptional responses to serum exposure are associated with sensitivity and resistance to antibody-mediated complement killing in invasive *Salmonella* Typhimurium ST313

**DOI:** 10.12688/wellcomeopenres.15059.1

**Published:** 2019-04-25

**Authors:** Edna M. Ondari, Elizabeth J. Klemm, Chisomo L. Msefula, Moataz Abd El Ghany, Jennifer N. Heath, Derek J. Pickard, Lars Barquist, Gordon Dougan, Robert A. Kingsley, Calman A. MacLennan

**Affiliations:** 1 Swiss Tropical and Public Health Institute, University of Basel, Basel, Switzerland; 2 University of Basel, Basel, Switzerland; 3 Novartis Vaccines Institute for Global Health, Siena, Italy; 4 Wellcome Trust Sanger Institute, Hinxton, UK; 5 Malawi-Liverpool-Wellcome Trust Clinical Research Institute, Blantyre, Malawi; 6 Department of Microbiology, College of Medicine, University of Malawi, Blantyre, Malawi; 7 The Westmead Institute for Medical Research and Marie Bashir Institute for Infectious Diseases and Biosecurity, University of Sydney, Westmead, Australia; 8 King Abdullah University of Science and Technology , Thuwal, Saudi Arabia; 9 Institute of Immunology and Immuotherapy, University of Birmingham, Birmingham, UK; 10 Helmholtz Institute for RNA-based Infection Research , Würzburg, Germany; 11 Faculty of Medicine, University of Würzburg, Würzburg, Germany; 12 Department of Medicine, University of Cambridge Addenbrooke's Hospital Cambridge, Cambridge, UK; 13 Quadram Institute Bioscience, Norwich, UK; 14 Jenner Institute, Nuffield Department of Medicine, University of Oxford, Oxford, UK

**Keywords:** Invasive salmonellosis, serum resistance, antibody, complement, genome, transcriptome

## Abstract

**Background**: *Salmonella* Typhimurium ST313 exhibits signatures of adaptation to invasive human infection, including higher resistance to humoral immune responses than gastrointestinal isolates. Full resistance to antibody-mediated complement killing (serum resistance) among nontyphoidal *Salmonellae* is uncommon, but selection of highly resistant strains could compromise vaccine-induced antibody immunity. Here, we address the hypothesis that serum resistance is due to a distinct genotype or transcriptome response in *S*. Typhimurium ST313.

**Methods**: Six *S*. Typhimurium ST313 bloodstream isolates, three of which were antibody resistant, were studied. Genomic content (single nucleotide polymorphisms and larger chromosomal modifications) of the strains was determined by Illumina and PACBIO sequencing, and functionally characterized using RNA-seq, transposon directed insertion site sequencing (TraDIS), targeted gene deletion and transfer of selected point mutations in an attempt to identify features associated with serum resistance.

**Results**: Sequence polymorphisms in genes from strains with atypical serum susceptibility when transferred from strains that were highly resistant or susceptible to a strain that exhibited intermediate susceptibility did not significantly alter serum killing phenotype. No large chromosomal modifications typified serum resistance or susceptibility. Genes required for resistance to serum identified by TraDIS and RNA-seq included those involved in exopolysaccharide synthesis, iron scavenging and metabolism. Most of the down-regulated genes were associated with membrane proteins. Resistant and susceptible strains had distinct transcriptional responses to serum, particularly related to genes responsible for polysaccharide biosynthesis. There was higher upregulation of *wca* locus genes, involved in the biosynthesis of colanic acid exopolysaccharide, in susceptible strains and increased expression of *fepE*, a regulator of very long-chain lipopolysaccharide in resistant strains.

**Conclusion**: Clinical isolates of *S*. Typhimurium ST313 exhibit distinct antibody susceptibility phenotypes that may be associated with changes in gene expression on exposure to serum.

## Introduction

Invasive nontyphoidal *Salmonella* (iNTS) infections are estimated to cause ~3.4 million illnesses and over 680,000 deaths annually ^1^, with a majority of these cases occurring in sub-Saharan Africa ^1, 2^. Predisposing factors include co-morbidities such as malaria ^3– 6^, HIV co-infection ^7– 9^, young age ^10, 11^, and malnutrition ^12– 14^. A clonal genotype of *Salmonella enterica* serovar Typhimurium ( *S*. Typhimurium) of sequence type 313 (ST313) ^15^, dominates iNTS disease in sub-Saharan Africa and this clone frequently encodes multiple drug resistance ^16– 21^. These factors likely contribute to the relatively high prevalence of invasive disease in the region compared to that observed with nontyphoidal *Salmonella* in other parts of the world, where gastroenteritis dominates.

A prominent signature in the genomes *S*. Typhimurium ST313 isolates is genetic degradation, associated with gene deletion and the accumulation of pseudogenes, resembling that observed in host-restricted *Salmonella* serovars such as *S*. Typhi, the causative agent of typhoid fever ^15^. Nevertheless, the ST313 found in Africa are monophyletic, falling into two highly conserved clades known as clades or lineages I and II ^15^. Phenotypically, ST313 isolates of both clades broadly exhibit decreased enteropathogenicity ^16, 22^, hyper-dissemination from the intestine ^23^, hyper-resistance to phagocytic killing ^24^ and complement ^20, 25^, and altered multicellular behaviour ^17^.

The predilection for invasive disease exhibited by ST313 underscores an important role for an effective humoral response to NTS during extra-intestinal infection ^11, 26^. Antibodies mediate opsonisation and killing by phagocytic cells ^26^, and direct bacterial killing by complement during inter-macrophage spread ^11^. Vaccines eliciting these responses, therefore, are a potentially promising means of protecting individuals from developing iNTS disease.

Multiple, and possibly redundant mechanisms for evading antibody responses have been described for *Salmonella*. These include survival in phagocytes associated with virulence factors such as *Salmonella* Pathognicity Island (SPI-2) ^27^, and the production of proteins that actively degrade or inhibit complement proteins such as PgtE ^28, 29^, Rck ^30^, PagC ^31^. and TraT ^32^. Recently, a SNP was identified in the promoter of *pgtE* resulting in high expression of the PgtE virulence factor in lineage II African ST313 *S.* Typhimurium. PgtE increases degradation of factor B component of human complement, likely contributing to serum resistance of the ST313 pathovar of *S*. Typhimurium ^20^.

Lipopolysaccharide (LPS) O-antigen length and composition can also play a key role in bacterial survival during bloodstream infection, by shielding bacteria against direct complement attack and killing by blood phagocytes ^33– 35^. Subunit vaccines that can induce antibodies capable of directing complement-mediated killing are under development for the prevention of iNTS disease in sub-Saharan Africa ^36– 40^. The presence of variants that evade antibody-mediated killing conferred by a vaccine could undermine this strategy. Here we address the hypothesis that variation in genotype or transcriptional response to serum of clinical isolates of iNTS correlates with serum susceptibility.

## Methods

**Bacteria and culture conditions**. Six *S*. Typhimurium from a series of 329 iNTS isolates from bacteraemic children admitted to Queen Elizabeth Central Hospital, Blantyre, Malawi with defined susceptibility to complement-mediated bactericidal activity were used in this study. Bacteria were routinely maintained in normal (10 g/l) or low salt (5 g/l) Luria-Bertani broth (Invitrogen, catalogue numbers 12795027 and 12780052 respectively) and grown aerobically at 37°C. Where appropriate, derivative mutant strains were grown in LB media supplemented with 100 µg/ml of hygromycin, or 50 µg/ml of kanamycin (Sigma-Aldrich, catalogue numbers 10687010 and 11815032 respectively).

**Investigation of the stability of the serum susceptibility phenotype**. Suspensions of two isolates, D23005 (serum resistant) and D24545 (highly serum sensitive) were spread on Luria-Bertani (LB) agar plates at a density of approximately 500 cfu and grown overnight. 100 colonies of each isolate were then grown to log phase in LB broth and exposed to serum for three hours. Serum susceptibility phenotype was then determined using serum bactericidal assays (described below).

**Identification of single nucleotide polymorphisms**. Sequence read alignment and detection of single nucleotide polymorphisms (SNP’s) were performed using paired-end Illumina sequence data mapped to the reference genomes *S*. Typhimurium D23580 and SL1344 with SMALT. SNPs were identified using samtools mpileup v0.2.0 and filtered with a minimum mapping quality of 30 and quality ratio cut-off of 0.75.

**Transfer of point mutations by transduction**. Nonsynonymous polymorphisms were transferred into *S*. Typhimurium D23580 by co-transduction with a selectable marker. A kanamycin resistance cassette was inserted into intergenic regions within 1kb of each selected point mutation in the donor strains by homologous recombination as described previously ^41^, using primers designed to avoid disrupting gene promoters (Table S1, Extended data ^42^). Phage P22 lysates of the resulting derivative strains were then used to infect D23580, using the kanamycin marker to select for transductants. Colonies were screened for the transfer of the desired allele using specific PCR oligonucleotide primers (Table S1, Extended data ^42^) and confirmed by sequencing.

**Serum bactericidal assays**. Normal human serum was obtained from the blood of a healthy adult donor. Clotting was performed at room temperature within two hours of the blood draw. Serum was then separated from the clotted fraction by centrifugation and stored in aliquots at -80°C until use. Test *S*. Typhimurium strains were grown to mid-log phase by inoculating 100-fold dilutions of overnight cultures into fresh LB broth and grown aerobically for 135 minutes at 37°C. The bacteria were then washed twice with phosphate-buffered saline (PBS), and approximately 1×10 ^6 ^cfu/ml were incubated with serum for 3 hours at 37°C. Serum susceptibility was determined by the change in numbers of viable bacteria after 3 hours of serum exposure, using serial dilutions of the serum-bacteria mixtures plated on LB agar.

**Transposon mutant library construction and screening**. A derivative of the transposon EZ-Tn5 (Epicentre, Catalogue number MOD1503) was phosphorylated with polynucleotide kinase (New England BioLabs Inc.), and incubated with EZ-Tn5 transposases (Epicentre, catalogue number TNP92110) at 37°C for 1 hour to prepare the transposome, which was stored at -20ºC until ready for use. D23580 electrocompetent cells were mixed with 0.5 µL of transposomes and transformed by electroporation in a 0.2 cm cuvette (Bio-Rad Laboratories) using parameters (2.5 kV, 25 F and 200 Ω) in Gene Pulser Xcell Electroporation System (Bio-Rad Laboratories). Cells were immediately re-suspended in 1 mL of SOC medium (Invitrogen, catalogue number 1554034) and incubated at 37°C for 2 hr before being spread on LB agar supplemented with kanamycin (10 µg/mL). The D23580 library consisted of 6×10 ^5^ mutants. Stationary phase cultures were then prepared by 1/200 dilution of mutant library in LB broth, vortexed briefly, and incubated aerobically at 37°C overnight with shaking. Log phase cultures (input library) were then prepared by 1/100 dilution of the overnight culture in LB broth and 2.5 hours of incubation at 37°C (without venting), on a rocker plate at 20 rev/sec. Viable counts of the input library were determined by serial dilution in PBS and plating of a sample of the neat input culture. An aliquot of the input library was harvested by centrifugation at 6000 rpm for 5 minutes. The supernatant was decanted, and the pellet washed three times in PBS (pH 7.4), then resuspended in PBS to a final concentration of 1×10 ^8^/ml bacteria. Serum bactericidal assays were performed as described above but using pooled serum from 10 healthy Malawian adults. After 180 minutes, the entire serum-bacteria suspension was transferred to 25 ml LB broth, vortexed and incubated overnight aerobically 37°C, to enable the surviving library to outgrow killed components. Viable counts were determined from the output library, and the remaining output culture pelleted at 6000 rpm for 20 minutes at 4°C, the supernatant removed, and frozen at -80°C until ready for use.

**Transposon directed insertion site sequencing (TraDIS)**. Genomic DNA (gDNA) of the initial (input) and selected (output) mutant pools was extracted using Qiagen Genomic-tip 100/G (Qiagen, Hiden, Germany, catalogue number 10043). Approximately 2µg of gDNA was fragmented to insert size of ~300 bp using E220 Evolution Somicator (Covaris). The fragmented DNA was end repaired, A-tailed and adapter-ligated using Illumina DNA fragment library preparation kit (New England BioLabs Inc., catalogue number E6177) according to the manufacturer’s instructions. The libraries were then enriched by 10-20 PCR cycles using a transposon-specific forward primer and a reverse primer that included Illumina flow cell binding sites as described previously ^43^. The enriched libraries were purified using the Agen court AMPure XP beads (Beckman Coulter, catalogue number A63882) and quantified on an Agilent DNA1000 chip (Agilent Technologies, Catalogue number 5067-1504) as per the manufacturer’s instructions. The libraries were sequenced on a HiSeq2000 Illumina platform as single-end reads of 100bp. The reads were processed using the Bio::TraDIS toolkit v1.132190 ^44^. Briefly, reads were filtered for 10 bases matching the expected transposon tag sequence. Filtered reads were then mapped against D23580 reference genome (GenBank accession number FN424405) using SMALT version 0.7.2, and transposon insertion sites and insertion indices (obtained by dividing the number of unique insertion sites by the gene length) were determined for every gene. Genes with read counts of less than 20 in all conditions were excluded from the analysis. Log _2_ fold changes and significance of changes in mutant abundance before (input; the basic mutant pool) and after selection (output; biological replicates of mutant pool screened in the presence of bactericidal serum) were calculated using edgeR v3.4.0 after normalization using TMM method ^45^.

**Targeted gene deletion in D23580**. Deletion of genes in *S*. Typhimurium D23580 was performed by recombinase-mediated allelic exchange as described previously ^41^. PCR fragments were made by amplifying the kanamycin resistance gene from plasmid pKD4, with each primer consisting of 50-mer homology arms flanking each targeted gene such that the entire open reading frame was deleted, and 20 nucleotides priming pKD4 at the 3’ends (primers in Table S1, Extended data ^42^). A second round of PCR using gel-purified products from the first reactions as templates was performed to eliminate pKD4 from the material used for electroporation. Recombinase-proficient D23580 bacteria harbouring the pSIM18 helper plasmid were transformed using approximately 1 µg of PCR product. Transformants were then selected on LB plates with kanamycin, and the gene replacement verified by screening colonies using PCR from extracted genomic DNA and primers specified in Table S1 (Extended data ^42^). Culture of transformants at 42°C (non-permissive temperature for pSIM18 replication), and isolation of kanamycin resistant and hygromycin sensitive colonies by replica plating was used to cure and verify loss of pSIM18.

**PacBio sequencing and assembly**. To prepare high molecular weight genomic DNA, log-phase cultures of each strain were prepared in 10 ml of LB broth as described above. Each culture was then centrifuged and the pellets resuspended in 2 ml of 25% (w/v) sucrose in TE buffer (10 mM Tris, 1 mM EDTA, pH 8.0). 5 mg of lysozyme (Roche, catalogue number 10837059001) was then added to each suspension, and incubated for 45 minutes at 37°C. 2 mg of proteinase K (Roche, catalogue number P4850), 300 μg of RNase A (Roche, catalogue number 10109169001), EDTA (pH 8.0) at a final concentration of 0.02 M, and 250 μl of 10% Sarkosyl NL30 (BDH/Fisher, catalogue number BP234-500) were added to the suspensions, then left on ice for 2 hours. Lysates were placed on a 50°C block overnight, then reconstituted to 5 ml using TE buffer. Samples were mixed with an equal volume of phenol:chloroform:isoamyl alcohol (25:24:1) in 15 ml phase-lock gel tubes (Eppendorf, catalogue number 0032005250), centrifuged for 15 minutes at 4000rpm, and the aqueous phases transferred to a fresh tube, and repeated three times. The aqueous phase was mixed with 2.5 volumes of 100% ethanol and incubated at -20°C for 1 hour to precipitate the DNA. The DNA was separated from the ethanol by centrifugation, washed with 70% ethanol, resuspended in TE buffer, and stored at -80°C until use.

PacBio sequencing was performed on a PacBio RS sequencer. Sequence reads were assembled using HGAP v3 ^46^ of the SMRT-Analysis software v2.3.0 ^47^. Sequence coverage for picking the minimum fragment length for assembly was set to 30, and the approximate genome size was set to 3 Mbp. The assembly was circularized using the pre-assembled reads in Circlator v1.1.3 ^48^. The circularized assembly was then improved using the PacBio RS_Resequencing protocol and Quiver v1 of the SMRT analysis software v2.3.0 ^47^.

**RNA sequence analysis**. Log-phase (OD _600 _0.2, determined using an Ultrospec 10 spectrophotometer, Amersham Biosciences) cultures of each strain were prepared in 10 ml of LB broth as described previously. Bacteria were resuspended to an OD _600_ of 2.0. 100 μl of this suspension was then inoculated into 900 μl of LB and 900 μl of normal human serum (to a final concentration of approximately10 ^8 ^cfu/ml) in 2ml microcentrifuge tubes. The tubes were incubated in a horizontal position with shaking at 60rpm at 37°C for 10 minutes, after which the bacteria were pelleted by centrifuging at 13000 rpm for 2 minutes, and the media/serum discarded. The pellets were resuspended in 1ml of ice-cold PBS. RNA was immediately stabilized as described previously ^49^, by adding 400μl (20%v/v) of a cold mixture of 100% ethanol and phenol saturated with 0.1 M citrate buffer at pH 4.3 (Sigma, catalogue number P4862) (19:1 v/v) to each sample and incubating on ice for 30 minutes. Extractions were performed using the FastRNA Pro Blue Kit (MP Biomedicals, catalogue number 116025050) according to the manufacturer’s protocol. Genomic DNA was removed from the samples using Turbo DNase (Ambion, catalogue number AM2238) with 4% v/v of RNAsecure RNase Inactivation Reagent (Ambion, catalogue number AM7005) added to each sample during DNase treatment to prevent RNA degradation. RNA was then purified using phenol-chloroform, with final elution in DEPC-treated water. Quality and concentration were checked by using a 2% agarose gel, and the Agilent 2100 Bioanalyzer RNA Nano protocol. Samples were then stored at -80°C until use. Extractions were performed in three biological replicates. RNA-seq was performed using an Illumina HiSeq 2000 sequencer, yielding 9-23 million reads per library from 100 cycles of paired-end sequencing (Sequencing and mapping statistics are in Table S2, Extended data ^42^). Sequence reads from each sample were mapped against the *S*. Typhimurium D23580 genome (Genbank accession number: FN424405). The reference was indexed, and the reads aligned using default parameters, with read trimming quality threshold set to 15 (q=15) and maximum insert size adjusted per library as the maximum requested fragment size of the sequencing library, implemented using BWA v0.7.12 ^50^. Expression values were computed from the read alignments to the coding sequence to generate the number of reads mapping ^51, 52^, including only reads with a mapping quality score of 10. Output spreadsheet files with features previously described ^53^ were obtained for each sample. Total read counts for each gene in every sample were used, with zero values in the data thresholded to 1. Principal Component Analysis (PCA) plots of normalized counts were used to identify samples that did not cluster with their replicates, and one sample (run accession ERR731338) was excluded from subsequent analysis. Data normalization, filtering, and calculation of fold changes were performed using DESeq2 (v.1.4.5) ^54^, and heatmaps and cluster dendrograms created using the *heatmap.2* function of the *gplots* package (v.2.13.0), all implemented in R v.3.1.3.

**Sequencing data accession number**. The RNA-seq and PacBio data generated in this study were submitted to the European Nucleotide Archive, available under study accession number ERP005455.

**Ethical approval**. Ethical approval was from the College of Medicine Research and Ethics Committee, University of Malawi (Protocol Number P.05/06/388). Peripheral blood samples were obtained following written informed consent from each donor.

## Results

**Figure 1.**
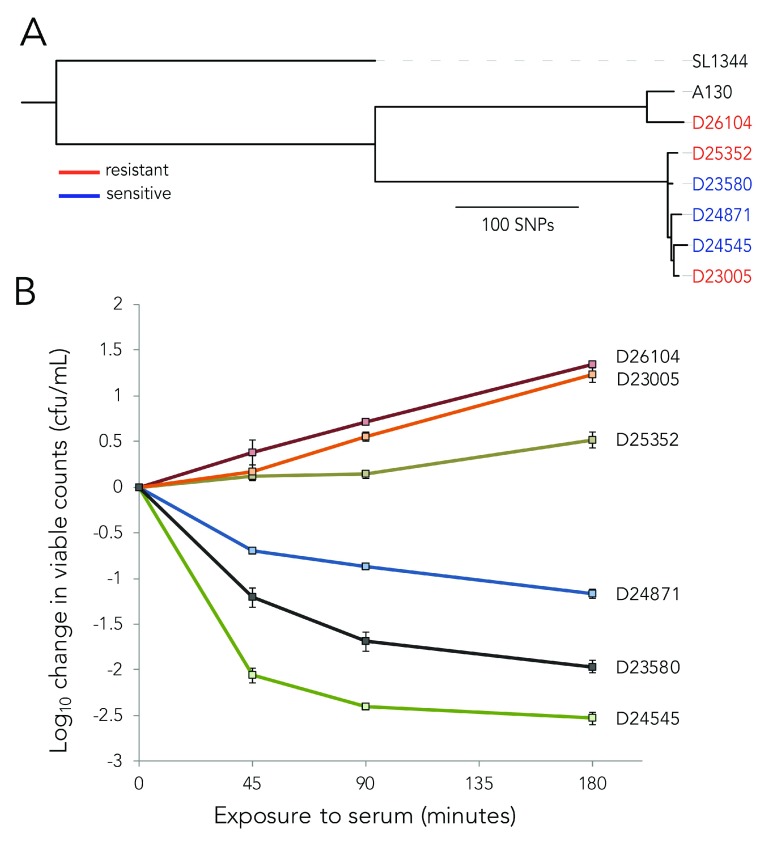
Characteristics of the six NTS isolates. **A.** Phylogenetic tree representing the relationships between the six NTS isolates studied. **B**. Fold change in bacterial counts following 3 hours of exposure to immune serum from a healthy adult donor. Each isolate was incubated at 37°C for 180 minutes with sampling at 45, 90 and 180 minutes. Data represent mean +/- standard error of two independent experiments, performed in triplicate.

***S*. Typhimurium isolates exhibit a stably inherited serum susceptibility phenotype.** We have noted that even phylogenetically highly related ST313, that vary by only a few SNPs, can exhibit reproducible differences in their resistance to serum killing. Thus, in order to investigate this further we selected five isolates of lineage II (D23580, D24545, D23005, D25352, D24871) and two (A130 (a reference isolate), D26104) of lineage I with different serum susceptibilities for detailed genotypic or phenotypic analysis. Two of these isolates, D23580 and D24871, were moderately susceptible to killing in normal serum. One isolate, D24545, was highly susceptible, while the other three had net growth after three hours of incubation in the same immune serum ( Figure 1 A and B). To investigate if serum susceptibility was a stably inherited phenotype during culture, we performed serum killing assays on 100 independent colonies each of a serum resistant (D23005) and susceptible (D24545) isolate. No obvious differences in phenotype for either isolate was observed (Figure S1, Extended data ^42^), therefore high frequency changes were considered unlikely to account for the observed variation in serum sensitivity.

**Complete genome analysis of the iNTS isolates with different serum susceptibility**. The genetic basis for variation in serum susceptibility between ST313 isolates is unknown. Therefore, to facilitate detailed genetic comparisons, we determined the complete genome sequence of each isolate using a combination of Illumina-generated short reads and PacBio long-read sequences ^55^. No major alterations in chromosome arrangement were apparent in any isolate using D23580 as the reference (Figure S2A (Extended data ^42^) and 2B). For example, there were no large chromosomal inversions and no major deletions involving multiple or single genes. The *traT* and *rck* genes, two known serum resistance genes, were both intact and had 100% sequence identity in all the six isolates. Similarly, no difference, other than single SNPs were found in the 88kb p14-95A plasmid between D23580 and other isolates was observed (Figure S2C, Extended data ^42^). Differences between ST313 clade I and isolates from clade II reflected lineage-specific genome evolution reported previously ^56^ ( Figure 1) (Figure S2, Extended data ^42^). We therefore concluded that gene flux and major genome rearrangements do not obviously correlate with serum susceptibility phenotype.

To investigate whether nucleotide substitutions (SNPs) or small indels impact the degree of susceptibility to serum killing, SNPs in gene coding and promoter regions were analysed in five lineage II isolates with D23580 as the reference ( Figure 1A). A total of 52 SNPs were identified (Table S3, Extended data ^42^), confirming the high genetic similarity. Of these, 13 SNPs resulted in predicted nonsynonymous amino acid substitutions that were unique to either the highly sensitive (D24545) or one of two resistant isolates (D25352 and D23005) ( Table 1).

**Table 1.** Polymorphisms occurring in clade II strains with atypical serum susceptibility. Only SNPs occurring in gene-coding regions and unique to each strain/phenotype are includeed. SNPs from *acrB*, *pepP*, *ispD*, uxu *B*, *uxuR*, and *STMMW_29841* genes were transferred to D23580 and tested for their impact on resistance or sensitivity to serum killing by normal human serum.

Position in D23580	Systematic Gene ID	Gene name	Description	D23580 base	SNP base	D24545 (highly sensitive)	D23005 (resistant)	D25352 (resistant)	AA CHANGE
570516	STMMW_05451	*acrB*	acriflavin resistance protein B	C	T	T	.	.	G796S
1200631	STMMW_11271	*agp*	glucose-1-phosphatase precursor, secreted	T	C	C	.	.	L322P
1264174	STMMW_11931	*rne*	ribonuclease E	G	T	T	.	.	R1062S
4277479	STMMW_40091	_	alcohol dehydrogenase	T	C	C	.	.	K78E
4390204	STMMW_41061	*rpoB*	DNA-directed RNA polymerase, beta-subunit	G	A	A	.	.	D516N
4783703	STMMW_44531	*uxuR*	uxu operon transcriptional regulator	G	A	A	.	.	W221STOP
3211805	STMMW_30181	*pepP*	proline aminopeptidase II	C	A	A	.	.	E79D
3064062	STMMW_28931	*ispD*	2-C-methyl-D-erythritol4- phosphate cytidylyltransferase	G	T	.	T	.	R128W
3175291	STMMW_29841	_	probable amino acid transport protein	T	G	.	G	.	S255L
1947840	STMMW_18471	_	putative membrane protein	T	A	.	.	A	D181E
1875804	STMMW_17771	_	conserved hypothetical protein	G	A	.	.	A	R349W
1966357	STMMW_18691	*pykA*	pyruvate kinase A	G	A	.	.	A	G124S
3289745	STMMW_30961	*uxuB*	D-mannonate oxidoreductase	A	C	.	.	C	N361T

**Figure 2.**
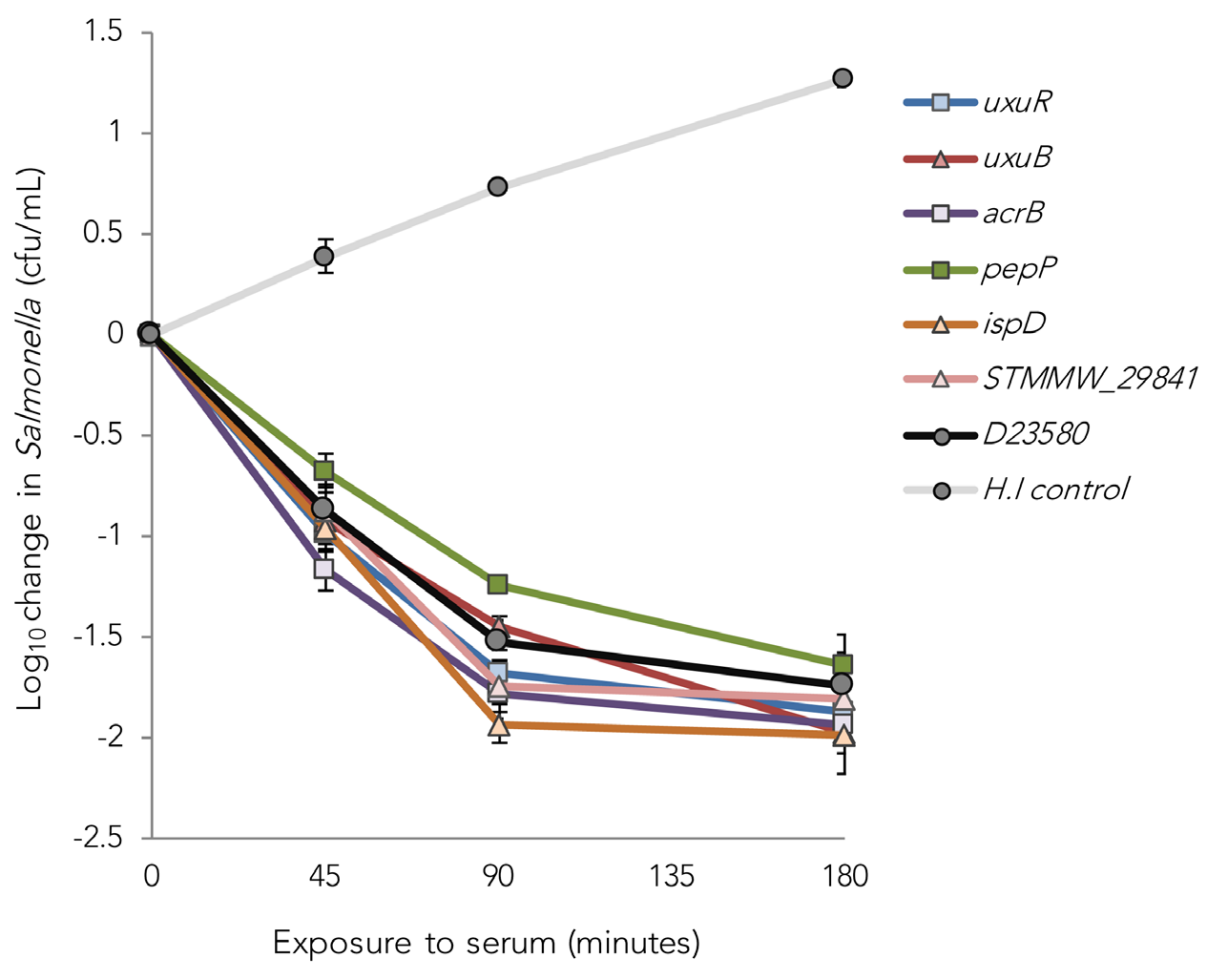
Relative serum sensitivity of D23580-derived strains with nonsynonymous polymorphisms transferred from serum resistant (D23005 and D25352) and susceptible (D24545) *Salmonella* Typhimurium strains. Each strain was exposed to immune human serum at 37°C for 180 minutes with sampling at 45, 90 and 180 minutes. Squares represent strains with alleles from D24545, triangles from the resistant strains. Data represent means of two independent experiments, performed in triplicate. Error bars: Standard Error. HI control: Heat-inactivated control (Wild-type D23580 exposed to heat-inactivated serum).

We tested whether these individual nonsynonymous SNPs accounted for the altered serum susceptibility phenotype by introducing them into D23580. Three of the SNPs (in *acrB*, *uxuR*, and *pepP*) were specific to D24545 which is highly susceptible to serum killing and three were present in resistant isolates D25352 or D23005 ( *uxuB*, *ispD*, and *STMMW_29841*). All of the recombinant D23580 derivatives exhibited similar sensitivity to wild type D23580 at all sampled time points, indicating that these polymorphisms had no detectable impact on serum susceptibility ( Figure 2).

For functional analysis of the 52 SNPs occurring in clade II isolates, we compared RNA-seq and TraDIS data corresponding to the regions where these SNPs occur. No significant changes, either in the viability of the corresponding transposon mutants or on the relative levels of transcription were observed (Table S3, Extended data ^42^). D26104, a lineage I isolate, had significantly diminished expression of *pgtE* when compared to reference isolate D23580, consistent with lacking the promoter mutation known to increase serum resistance in lineage II isolates ^20^ (Table S8, Extended data ^42^). Despite this, D26104 is a highly serum resistant isolate, suggesting an alternative mechanism of resistance in this isolate that is unlikely to be associated with the *pgtE* gene.

**Identification of genes required for full serum resistance of** *S* **. Typhimurium D23580.** By a complementary approach, we identified genes required for serum resistant ST313 using a whole genome functional screen with a saturating Tn5 transposon insertion library in D23580. The number of Tn5 transposon insertion mutants in each gene before and after exposure to serum for 180 minutes was determined by transposon directed insertion site sequencing (TraDIS). We classified genes as ‘serum resistance determinants’ if their disruption resulted in decreased viability in serum and therefore under-representation at the final timepoint. Under-representation was determined by a reduction in mapped read counts (relative to the library prior to serum exposure) of at least 2-fold and with an adjusted p-value of <0.05 or less. Similarly, we defined genes as ‘serum susceptibility determinants’ if their disruption resulted in increased viability in serum and therefore over-representation at the final timepoint. We identified 555 serum resistance determinants and 82 serum susceptibility determinants (Complete results in Table S4, Extended data ^42^).

**Figure 3.**
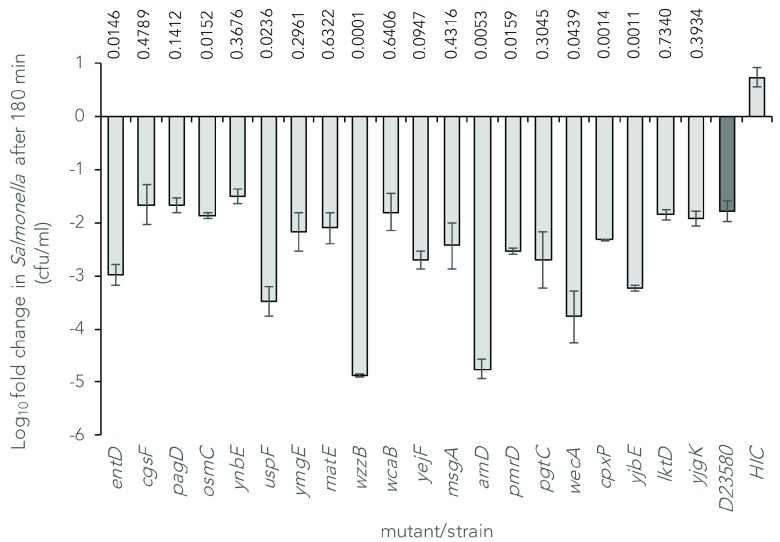
Susceptibility of D23580 mutants to the bactericidal activity of normal human serum relative to the wild-type strain. Bars show the mean log _10 _fold change +/- standard error of the mean in viable counts of twenty *S*. Typhimurium D23580 mutants following exposure to serum for 180 minutes. HIC: Heat-inactivated control (Wild-type D23580 exposed to heat-inactivated serum). P values above the bars represent t-test probabilities of pairwise comparisons of fold changes between each mutant strain and the wild-type strain.

To validate the transposon insertion library screen, we constructed defined mutations in D23580 by independently replacing 20 of the serum-resistance determinants with the *aph* gene conferring kanamycin resistance, by allelic exchange. The deleted genes were from a broad range of functional categories. Nine of the mutants tested ( *entD*, *osmC*, *uspF*, *wzzB*, *arnD*, *pmrD*, *wecA*, *cpxP* and *yjbE*) had significantly increased susceptibility to serum compared to wild–type D23580 ( Figure 3).

**Figure 4.**
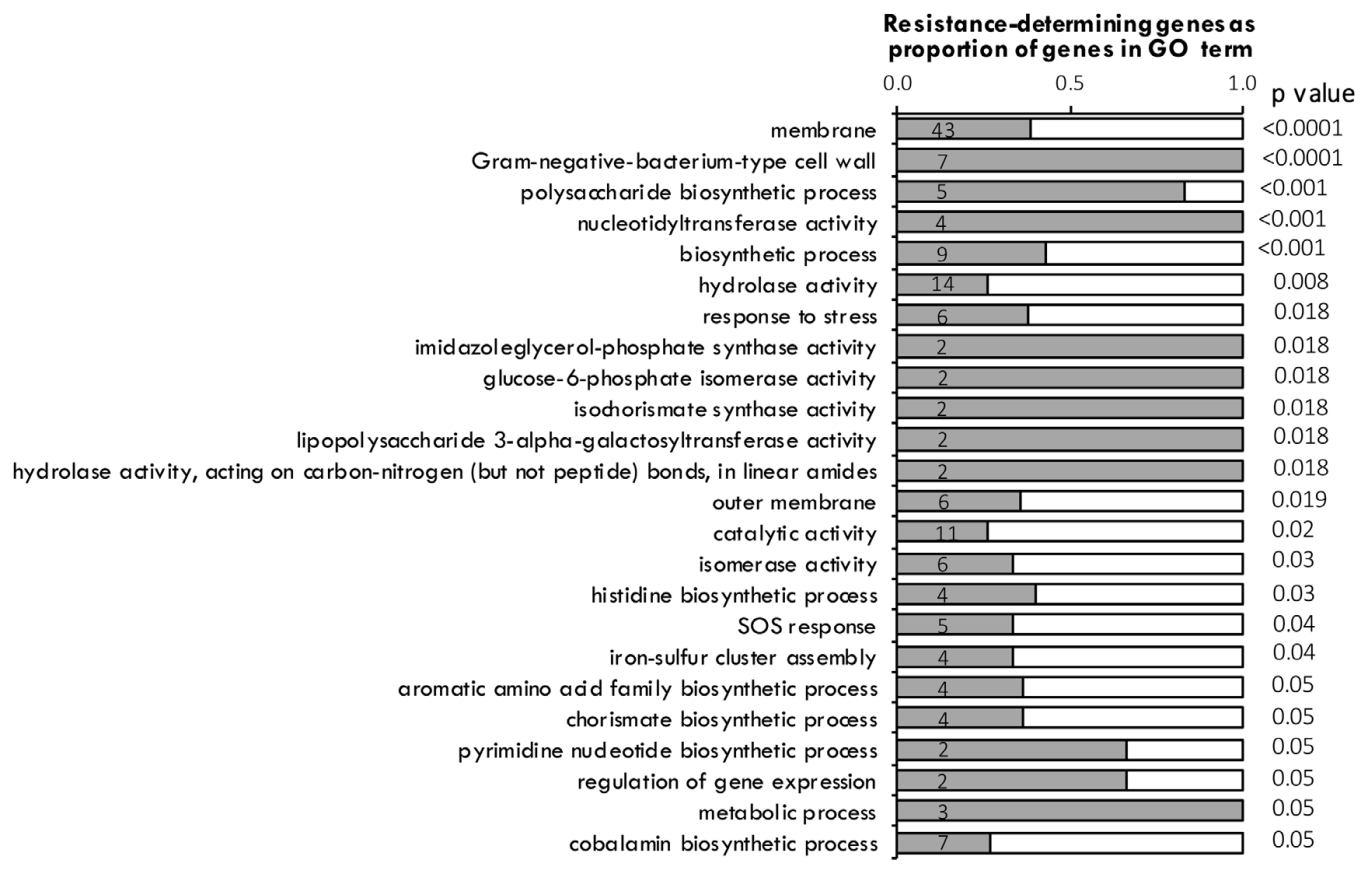
Significantly enriched gene ontology (GO) terms for 555 serum resistance-determining genes in D23580. Genes included in the analysis were those whose corresponding mutants had a fold change of 2 ^-2 ^or lower from comparisons of input and output mutant libraries identified by TraDIS. P values are hypergeometric probabilities calculated using the *phyper* function in R. Comparisons were made using the number of genes associated with each term in the gene set versus the total number of genes associated with each term in the genome, using *Salmonella* Typhimurium LT2 GO annotation ( http://www.uniprot.org/proteomes/UP000001014). Numbers within the bars represent the total number of genes in the essential gene set associated with each term.

Gene ontology (GO) terms associated with proteins encoded by serum-resistance determinants (Table S5) identified significant enrichment of membrane components, particularly genes responsible for membrane and extracellular polysaccharides such as lipopolysaccharide (LPS) and enterobacterial common antigen (ECA) synthesis (33 genes) ( Figure 4). Also, genes involved in iron transport, iron ion binding, or iron-sulfur cluster assembly binding were significantly represented (30 genes total), consistent with the iron-limiting conditions of serum contributing to diminished bacterial viability ( Figure 4, Table S5 (Extended data ^42^)). 

**Distinct transcriptional response is associated with serum susceptibility.** To investigate whether variation in gene expression was associated with serum sensitivity in isolates with differential degrees of sensitivity, we analysed changes in transcription from bacteria exposed to normal human serum for 10 minutes relative to standard laboratory conditions (mid log culture in LB). This identified 170 genes as differentially expressed in all six isolates, 88 of which were up-regulated and 82 down-regulated (Tables S6, S7). A significant proportion (49%) of the up-regulated genes included ones involved in iron scavenging and iron-associated metabolism, including *fhu, ent, fep, suf, iro, exb, feo,* and the *sit* operon (Table S6, Extended data ^42^). The second most abundant group (12.5%) consisted of genes involved in colanic acid biosynthesis, including the *rcsA* regulator (Table S6, Extended data ^42^). A majority of the down-regulated genes (55%) were associated with the bacterial envelope (Table S7, Extended data ^42^). Notably, *S*. Typhimurium serum resistance genes *rck*, *traT*, and *pgtE* ^20^, were not significantly up-regulated in our experiments (Table S8, Extended data ^42^).

**Figure 5.**
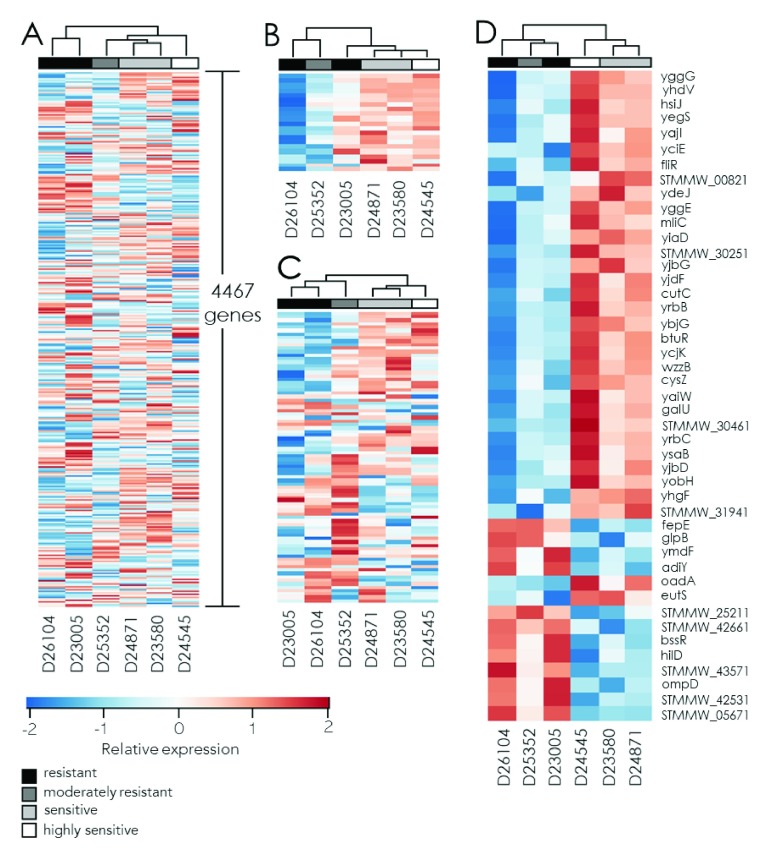
Relative gene expression in six invasive *Salmonella* Typhimurium strains in response to serum exposure. Heat maps indicate log _2_ fold changes in transcript abundance following a 10-minute growth in serum compared to log-phase cultures in LB. Patterns of transcriptional responses are clustered and their relationship indicated by dendrograms. The intensities of each cell colour represent the deviation (Z-score) of each strain from the average fold change (centered to zero, represented with white on the heatmap) per gene for higher (red) or lower (blue), for all chromosomal genes ( **A**), genes in the *wca* (colanic acid biosynthesis) locus ( **B**), LPS biosynthesis genes ( **C**), or genes satisfying a t-test cut-off of ≤0.05 and a ratio of ≥2 ( **D**).

We also identified transcriptional patterns that distinguished the isolates by serum susceptibility phenotype. Unsupervised clustering of the six *S*. Typhimurium strains based on the magnitude of change in expression for all chromosomal genes revealed a pattern that approximately grouped the isolates by relative serum sensitivity ( Figure 5A). The transcriptional response to serum killing of D26104 and D23005 that had the greatest resistance to serum killing were most similar to one another, D25352, D24871 and D23580 that were moderately resistant or sensitive clustered, whilst the extremely susceptible strain D24545 had a distinct profile that was nonetheless most closely related to other susceptible strains. Since both colanic acid and LPS polysaccharides are known to be associated with serum resistance in pathogenic Enterobacteria ^34, 35, 57^ we investigated the transcriptional profile genes involved in their biosynthesis in the six strains ( Figure 5B and C). A modest level of clustering of resistant and susceptible strains was also observed for genes involved in colanic acid biosynthesis and to a greater degree LPS biosynthesis. Notably, despite significant induction of a majority of the *wca* locus genes in all the strains (Table S6, Extended data ^42^), elevation of transcription of these genes was higher in serum susceptible than serum resistant strains ( Figure 5B).

We also identified genes that exhibited a significant difference in mean expression greater than 2-fold in the three resistant strains compared to the three susceptible strains, (p<0.05). A total of 45 genes had a ratio of 2 or more from comparison of means of either group, most of which are associated with putative or uncharacterized proteins ( Figure 5D). Genes more highly expressed in response to serum in sensitive compared with resistant strains included *yrbC*, *yiaD*, which is a putative outer membrane ( *ompA*) family protein, *yibD*, which is expressed in response to phosphate starvation, *yggE*, an oxidative response protein, heat shock protein *hslJ*, homeostasis protein *cutC*, lysozyme inhibitor *mliC*, flagellar biosynthetic protein *fliR*, metabolic proteins *eutS*, *btuR*, *galU* and *odaA*, *cysZ* transporter, *yjbG*, which increases resistance to bacitracin, and *yaiW*, a putative surface-exposed outer membrane lipoprotein. 12 genes were more highly up-regulated in response to serum in resistant compared with sensitive strains, and included *glpB*, porin protein *ompD*, *hilD*, a transcriptional regulator for multiple virulence genes, and *bssR*, which is induced in biofilms.

Notably, two LPS O-antigen genes, *wzzB* and *fepE* exhibited distinct responses to serum in resistant and susceptible strains. While the O-antigen polymerase *wzzB* was on average more highly up-regulated in serum-susceptible strains relative to serum-resistant ones, *fepE*, which modulates expression of very long O-antigen chains ^34^, was on average 2-fold more highly expressed in serum-resistant strains than susceptible strains ( Figure 5D).

## Discussion

*S*. Typhimurium ST313 isolates that are highly resistant to serum killing have been identified raising the possibility that these may be the basis of escape mutants capable of evading vaccine-mediated antibody and complement killing. To better understand this problem, we investigated the molecular basis of resistance to antibody-mediated complement killing among iNTS isolates. First, we analysed the genomic content of six bloodstream isolates with differing levels of resistance, to identify SNPs or larger modifications that may have an impact on serum resistance. By this means, we were unable to find a basis for the differences in resistance.

TraDIS and transcriptomic profiling analyses revealed the repertoire of genes involved in survival and growth in serum in these *S*. Typhimurium ST313 isolates. Two major functional groups of differentially selected or expressed genes following serum exposure were identified: genes involved in nutrient scavenging, mainly the uptake and assimilation of iron, and genes involved in the synthesis of bacterial envelope components, particularly of extracellular polysaccharides. Several genes significantly up-regulated on exposure to serum, including *ent*, *iro*, *sit*, *feo* operons, *tonB*, *exbB* and *exbD* were essential for normal levels of viability in serum. For example, deletion of the *entD* gene in D23580 resulted in 40-fold lower viability than wild-type following 3 hours of exposure ( Figure 3). Genes classically associated with serum resistance in *S*. Typhimurium, such as *rck*, *traT*, *pgtE*, however, were not significantly up-regulated at the time point investigated (Table S6, Extended data ^42^). 

The bacterial membrane and its associated components are important for viability in serum. Whilst LPS genes were not significantly up-regulated during serum exposure, we did observe differences in expression patterns of LPS genes between the serum resistant and susceptible strains. In particular, the expression of O-antigen polymerase genes *wzzB* and the very long O-chain determinant *fepE*, were significantly different among these strains, with an average two-fold higher expression of *fepE* detectable within 10 minutes in resistant compared with susceptible strains as determined by RNAseq. Differences in the induction of these two genes resulting in preferential polymerization of LPS into trimodal (including very-long) rather than bimodal (with long) O-chain lengths confer resistance to antibody-dependent complement-mediated serum bactericidal activity in *S*. Typhimurium ^34^.

Increased expression of *wca* locus genes, responsible for the synthesis of colanic acid exopolysaccharide was observed. Twenty genes comprise this operon in *S*. Typhimurium ^58^, ten of which were differentially expressed in all six strains, along with *rcsA*, which regulates their expression. Rcs-associated up-regulation of colanic acid genes in response to serum has been observed in pathogenic *E. coli* ^57, 59^, and is also induced by exposure to antimicrobial peptides in *S*. Typhimurium ^60^. Expression of colanic acid is thought to mediate serum resistance either through formation of a barrier against lysis by the membrane attack complex ^57^ or preventing complement activation by limiting access to LPS ^58^. Although we found that colanic acid biosynthetic genes increased expression on exposure to serum, induction was greatest in susceptible strains. Furthermore, Tn5 insertions in colanic acid biosynthetic genes were not counter-selected in the TraDIS screen, and a null *wcaB* mutant was not more sensitive to antibody killing than the wild-type strain.

The relative lower induction of colanic acid biosynthetic gene in resistant strains may be linked to the higher increase in *fepE* expression in these strains. FepE is a positive regulator of very long LPS O-antigen chain associated with enhanced serum resistance ^34^. In *S*. Typhi, *fepE* is a pseudo gene ^61^, and very long LPS O-antigen chain interferes with the expression of the Vi exopolysaccharide capsule ^61^. Lower expression of colanic acid biosynthesis genes may therefore facilitate the elaboration of very long LPS O-antigen chain and enhance serum resistance.

The large-scale genome-wide approaches used in this study provided a robust platform for investigating the full complement of genes necessary for survival of *S*. Typhimurium in serum. As with most high-throughput assay systems, some caveats apply. For instance, with mutant libraries, it is not possible to distinguish between mutations associated with an overall decrease in bacterial viability or fitness over ones specifically necessary for complement resistance during selection in serum. Additionally, since the environmental signals *ex vivo* or *in vitro* are not identical to those *in vivo*, the information from the assays may not fully recapitulate the *in vivo* response to antibody-dependent complement killing. However, complementary results from the multiple approaches used in this study, mitigate these limitations to an extent.

We have defined gene functional classes and specific genes associated with serum resistance in six *S*. Typhimurium bloodstream isolates. Investigation of the transcriptional response of resistant and sensitive isolates highlighted putative factors responsible for the diverse serum sensitivity phenotypes exhibited by isolates of *S*. Typhimurium ST313. While susceptibility to antibodies was not associated with an identifiable genotype, distinct transcriptional responses were evident in resistant and sensitive isolates, in particularly transcriptional response of genes associated with surface polysaccharides. It is hoped that this study will provide a basis for further research into modes of escape from natural and vaccine-induced immunity among invasive *Salmonella* strains.

## Data availability

### Underlying data

Transcriptional profiling of Salmonella Typhimurium during serum exposure, Accession number ERP005455: http://identifiers.org/ena.embl:ERP005455

RNA-seq of S. Typhimurium bloodstream isolates following exposure to serum versus LB broth controls, Accession number: E-MTAB-7449: http://identifiers.org/arrayexpress:E-MTAB-7449

Open Science Framework: Genome and Transcriptome Analysis of invasive African S. Typhimurium isolates. https://doi.org/10.17605/OSF.IO/TM5WN ^42^

This project contains the following underlying data:

- Count Data_Serum Bactericidal Assays.xlsx (Raw counts from bactericidal assay)

### Extended data

Open Science Framework: Genome and Transcriptome Analysis of invasive African S. Typhimurium isolates. https://doi.org/10.17605/OSF.IO/TM5WN ^42^

This project contains the following extended data:

- Fig_S1.pdf (Susceptibility of 100 clones of S. Typhimurium ST313 strains, D23005 (serum resistant) and D24545 (serum susceptible) following exposure to normal human serum for 3 hours)
- Fig_S2.pdf (Comparisons of chromosomes and major plasmids in six S. Typhimurium strains displaying different antibody sensitivities. Blocks represent pairwise blastn comparisons of A. chromosomes B. pSLT virulence plasmid and C. S. Typhimurium p14-95A from clade II strains. *p14-95A was not found in D26104. Comparisons were made using the Artemis Comparison Tool (ACT))
- Table S1_Primers Used in this study.docx (list of study primers)
- Table S2_Mapping Statistics.csv (RNA-seq mapping statistics)
- Table S3_SNP Analysis.xlsx (Extended analysis of single-nucleotide polymorphisms in lineage II isolates)
- Table S4_D23580_TraDIS_Analysis.csv (D23580 TraDIS full analysis)
- Table S5_GO Terms associated with TraDIS serum resistance determinants.csv (GO terms associated with serum resistance determinants)
- Table S6_RNA_Seq_Upregulated.xlsx (Up-regulated genes determied by RNA-seq)
- Table S7_RNA-Seq_Downregulated.xlsx (Down-regulated genes determied by RNA-seq)
- Table S8_Expression of known S. Typhimurium serum resistance-associated genes.csv (Expression of serum resistance-associated genes *rck*, *traT*, and *pgtE*)

Data are available under the terms of the Creative Commons Attribution 4.0 International license (CC-BY 4.0).
