# Supplementary material for: Rapid transcriptional responses to serum exposure are associated with sensitivity and resistance to antibody-mediated complement killing in invasive Salmonella Typhimurium ST313
Source: Wellcome Open Res. 2019 Apr 25;4:74. [Version 1] doi: 10.12688/wellcomeopenres.15059.1 (PMC6560496; doi:10.12688/wellcomeopenres.15059.1)
Supplement: Peer review history

# Peer review: Reviewer response for version 1

**DOI:** 10.21956/wellcomeopenres.16429.r35440

**Published:** 2019-06-10

**Authors:** Mark A. Schembri

**Affiliations:** 1 School of Chemistry and Molecular Biosciences, The University of Queensland, Brisbane, Australia

This manuscript describes an analysis of 3 serum-resistant and 3 serum-sensitive S. Typhimurium ST313 bloodstream isolates using a combination of genome sequence analysis, TraDIS and RNA-seq. Several large datasets were generated and analysed. Based on genome analysis, no chromosomal modifications could be attributed to serum resistance or susceptibility. In contrast, the combination of TraDIS and RNA-seq analysis identified genes involved in exopolysaccharide synthesis, iron scavenging and metabolism that were required for resistance to serum.

 The manuscript is generally well written and my comments are all minor. Overall, I think this work represents a valuable contribution that will be of interest to other researchers in the field.

 Comments

- It is stated that phylogenetically related ST313 that vary by only a few SNPs can exhibit reproducible differences in their resistance to serum killing. Do the authors mean core SNPs? 
- Five isolates of lineage II (D23580, D24545, D23005, D25352, D24871) and two of lineage I (A130 and D26104) with different serum susceptibilities were studied. Are these strains of the same serotype?
- The traT and rck genes, two known serum resistance genes, were both intact and had 100% sequence identity in all the six isolates. Identity is used to compare amino acid sequences. Therefore, it would be more accurate to refer to the nucleotide sequences as 100% conserved. 
- It wasn’t clear if the TraDIS reads have been deposited to the SRA. Please clarify and if not provide accession numbers.
- The application of TraDIS and RNA-seq provides a powerful combination to understand serum resistance. I think the authors could discuss how the combining these two methods enhanced their overall findings.

I have read this submission. I believe that I have an appropriate level of expertise to confirm that it is of an acceptable scientific standard.

# Peer review: Reviewer response for version 1

**DOI:** 10.21956/wellcomeopenres.16429.r35647

**Published:** 2019-05-29

**Authors:** Manuela Raffatellu

**Affiliations:** 1 Division of Host-Microbe Systems & Therapeutics, Department of Pediatrics, University of California San Diego, La Jolla, CA, USA

Ondari *et al.* investigate mechanisms of serum resistance/sensitivity in six S. Typhimurium ST313 isolates. Sequence polymorphisms were identified, but their transfer to a strain with intermediate resistance did not affect serum resistance. TraDIS and RNAseq identified genes required for serum resistance, including those involved in exopolysaccharide synthesis, O-antigen length, colonic acid biosynthesis, and iron metabolism.

 Overall, this is an important study that aims to address key mechanisms of pathogenicity of African Salmonella isolates. The manuscript is clearly written, the experiments are well conducted, and the results are carefully interpreted and discussed.

1. One question that remains to be addressed is the mechanisms underlying the differences in gene expression between isolates that are serum resistant and the ones that are serum sensitive. Based on the authors’ data, it appears that the differences in the genomes are relatively minor, while differences in gene expression (e.g., Fig. 5) are quite dramatic. How do the authors explain these differences in gene expression, despite very similar genomes? Are there SNPs in the promoter of key regulators? Differences in the expression of key regulators? If the current data do not reveal differences in key regulatory mechanisms, then the authors should discuss possible mechanisms underlying the differences in gene expression between isolates.
2. One of the authors’ conclusions is that iron uptake genes are associated to serum resistance. Included in this list is the sit operon. Although initially identified as an iron transporter, sitABCD is primarily a manganese transporter. Also, the authors’ datasets list another manganese transporter, mntH. Thus, the authors data indicate that both iron and manganese uptake genes are associated to serum resistance. I suggest that the results and discussion sections are updated to include manganese transport genes (sit and mntH).

I have read this submission. I believe that I have an appropriate level of expertise to confirm that it is of an acceptable scientific standard.
